# Evaluation of non-invasive hemoglobin measurement in blood donors at a tertiary care hospital, Bangkok, Thailand

**DOI:** 10.1371/journal.pone.0348447

**Published:** 2026-05-07

**Authors:** Ratinan Dangwilailert, Somboon Lekmak, Duangtida Promlee, Tanyaporn Pongkunakorn, Parichart Permpikul

**Affiliations:** 1 Faculty of Medicine Siriraj Hospital, Mahidol University, Bangkok, Thailand; 2 Department of Transfusion Medicine, Faculty of Medicine Siriraj Hospital, Mahidol University, Bangkok, Thailand; 3 Research Department, Faculty of Medicine Siriraj Hospital, Mahidol University, Bangkok, Thailand; Shoklo Malaria Research Unit, THAILAND

## Abstract

**Background:**

Hemoglobin (Hb) assessment is crucial to prevent blood donation from anemic donors. Most measurements are invasive and painful; however, an alternative, non-invasive hemoglobin measurement is available. This study aimed to compare non-invasive and point-of-care invasive hemoglobin test results with those from an automated blood analyzer in blood donors.

**Methods:**

Qualified blood donors were enrolled at a tertiary care hospital in Bangkok, Thailand. Hemoglobin was initially measured by the non-invasive device (Rad-67 Pulse CO-Oximeter) and a point-of-care invasive hemoglobin screening device (Mission HemoPro). Participants with point-of-care invasive hemoglobin ≥ 12.5 g/dL were eligible to donate, and during donation, standard hemoglobin measurements were obtained using an automated analyzer, XN-550. Those who were ineligible with point-of-care invasive hemoglobin < 12.5 g/dL underwent additional blood sampling for hemoglobin measurement by an automated analyzer. Correlation and agreement between non-invasive, point-of-care, and automated hemoglobin measurements were assessed with Intraclass Correlation Coefficient (ICC) and Bland-Altman plots, while satisfaction was evaluated using a Likert scale.

**Results:**

Of 300 participants, 295 had complete data. Of these, 169 were male (57.28%). Average non-invasive, point-of-care, and automated hemoglobin levels were 14.38 ± 1.12 g/dL, 13.65 ± 0.70 g/dL, and 13.90 ± 1.16 g/dL, respectively. The ICC between non-invasive, point-of-care, and automated hemoglobin measurement was 0.600 (95% CI: 0.522–0.668) and 0.897 (95% CI: 0.872–0.957). The sensitivity of the non-invasive Hb measurement was poor for detecting anemic donors when the Hb cut-off was set at 12.5 g/dL. This method received significantly higher satisfaction than the routinely used invasive device.

**Conclusion:**

The non-invasive Hb measurement in blood donors showed moderate agreement with the standard test, but the sensitivity was poor when the cut-off hemoglobin was set at 12.5 g/dl. Since donor satisfaction was higher, this method may be used as an alternative screening tool, provided a higher Hb cut-off value is used.

## Introduction

Whole blood donation is a multi-step process that aims to collect blood from qualified, healthy donors. Evaluating prospective blood donors’ hemoglobin (Hb) levels is one of the most critical steps to ensure blood donor safety [1] and the quality of blood products. Currently, several screening methods are used, namely the copper sulfate gravimetric method, point-of-care hemoglobin measurement, and automated hematology analyzer-based hemoglobin measurement [2].

Automated analyzers provide precise Hb measurements, but they require venous sampling. With this method, donors undergo two venipunctures, one for Hb testing and the other for donation [2]. Therefore, it is not routinely used as a screening method. As for the copper sulfate gravimetric method, which is based on the principle that a drop of whole blood with adequate hemoglobin will sink when introduced into a copper sulfate solution of a specific gravity (1.053). This simple technique was used long ago, but studies disclosed poor performance [3]. At present, most blood banks, including ours, use point-of-care capillary (POC) hemoglobin measurement, which is quick and reliable. However, this method involves a finger prick, which some donors find more painful than venipuncture [4]. Also, studies showed variation of the results from the standard automated analyzer [5]. Over the past decade, non-invasive methods for measuring hemoglobin have been developed [2]. In Thailand, the Rad-67 Pulse CO-Oximeter (Masimo Corporation, Irvine, CA, USA), a non-invasive hemoglobin measurement using spectrophotometry, has been introduced. With the application of multiple light-emitting diodes (500–1400 nm), light is transmitted through the donor’s fingernail. Absorption of signals is measured based on blood-flow velocity (maximum light-intensity detection ≤ 25 mW) [6]. Studies on this test have been conducted in various settings, for example, perioperative units [7], critical care [8], antenatal care [9], non-pregnant women [10], and emergency rooms [11]. This method is convenient, quick, and non-invasive. However, its accuracy may be reduced in individuals with peripheral vascular abnormalities or circulatory disorders [6]. There was a study on non-invasive hemoglobin measurement in blood donors that revealed a substantial correlation between the non-invasive hemoglobin value and hemoglobin from the complete blood count (CBC), with an ICC of 0.69. The non-invasive technology used was occlusion spectrophotometry, a different technology from that used in our study [12].

A comparative study of hemoglobin measurement efficiency among non-invasive spectrophotometry device, point-of-care invasive device, and the standard automatic analyzer revealed that the point-of-care invasive device produced results more similar to the standard test than the non-invasive spectrophotometry one, with a correlation coefficient of 0.85 (95% CI [0.78–0.90]) and 0.77 (95% CI [0.67–0.84]), respectively [13]. Notably, the non-invasive spectrophotometry device yielded lower hemoglobin levels than the other two methods. Given its non-invasiveness, further studies are needed to assess the efficacy of the non-invasive spectrophotometry device before recommending its use in blood donation.

Therefore, at a tertiary care blood donation center, we conducted a study to compare Hb level obtained from the non-invasive device ([SpHb] Rad-67 Pulse CO-Oximeter [Masimo Corporation, Irvine, CA, USA]) and the point-of-care Hb measurement (HemoPro) with those from the standard automated blood analyzer (XN-550 [Sysmex Asia Pacific Pte Ltd., Asia Green, Singapore]). Additionally, satisfaction levels were compared between the two tests.

## Materials and methods

### Study population

We conducted a single-center study from September 15, 2024, to January 12, 2025, at the Blood Donation Center, Siriraj Hospital, Bangkok, Thailand. The study protocol was approved by the Siriraj Institutional Review Board (COA no. Si 606/2024). All participants provided written informed consent before enrolling in this study.

We enrolled blood donors aged 18 or older who met the national guidelines and provided written informed consent. Exclusions included apheresis donors, individuals with peripheral vascular issues such as Raynaud’s or Scleroderma, and those with nail enhancements, including extensions or artificial nails.

### Hb measurement

Three devices used for Hb measurement were examined: (1) a non-invasive device using the Rad-67 Pulse CO-Oximeter [Masimo Corporation, Irvine, CA, USA], (2) a point-of-care invasive device routinely used at the Blood Donation Center, the Mission® HemoPro Hemoglobin meter [ACON Laboratories, San Diego, USA], and (3) a reference method using an automated hematology analyzer, the XN-550 [Sysmex Asia Pacific Pte Ltd., Asia Green, Singapore].

First, the Hb level was measured using the Rad-67 by placing the ring finger of either the left or right hand on the device#39;s sensor and keeping it in place until the reading stabilized. The Hb level (Hb_Rad-67_) was then recorded.

Second, a capillary blood sample was obtained from the same finger used for the Rad-67 measurement, and hemoglobin was measured by the point-of-care device (HemoPro, referred to as Hb_Hemopro_).

Third, based on their Hb_Hemopro_ levels, participants were classified into the two groups for subsequent Hb measurement using an automated CBC analyzer (Hb_CBC_).

**Group 1**: Participants with Hb_Hemopro_ level ≥ 12.5 g/dL were considered eligible for blood donation. A 3 mL venous blood sample was collected from the diversion pouch during the pre-donation process and placed in a K3-EDTA tube for Hb_CBC_ analysis.

**Group 2**: Participants with Hb_Hemopro l_evels below 12.5 g/dL were classified as ineligible for donation, and these individuals underwent venipuncture to collect a 3 mL blood sample into a K3-EDTA tube for Hb_CBC_ analysis.

Participants with both Hb_Hemopro_ and Hb_CBC_ values < 12.5 g/dL were deferred from blood donation.

### Satisfaction measurement

Satisfaction was assessed based on duration, pain, and feeling safe with the test devices. Using a Likert scale, scores ranged from 1 (least satisfied) to 5 (most satisfied). Three domains were used: [1] duration – the time taken for the test, [2] pain – the pain experienced during the test, and [3] safety – the feeling of safety during the test. Participants who rated their satisfaction at levels 1–3 were classified as “not satisfied”, and those who rated their satisfaction at levels 4–5 were classified as meeting the “satisfied” criteria. The number of participants who provided ratings within the “satisfied” criteria across all three factors will be counted and compared.

### Statistical analysis

Data were recorded in Microsoft Excel, and the statistical analyses were performed using IBM SPSS Statistics version 29.0 (IBM Corp, Armonk, NY, USA). Demographic data were analyzed using descriptive statistics. Categorical data are presented as numbers and percentages, while quantitative data are presented as means and standard deviations (SD). The correlation between Hb_Rad-67_ vs Hb_CBC_ and Hb_Hemopro_ vs Hb_CBC_ was assessed using an Intraclass Correlation Coefficient (ICC) and displayed with a Bland-Altman plot, which shows the mean bias and 95% limits of agreement (LoA). Additionally, the consideration of the ICC for comparing variance caused by differences in measuring instruments follows the defined principles as ICC range as follows: excellent (ICC: 0.91–1.00), good (ICC: 0.76–0.90), moderate (ICC: 0.50–0.75), and poor (ICC < 0.50) [14].

We also calculated the HemoPro and Rad-67#39;s blood donor screening efficiency through sensitivity, specificity, positive predictive value, negative predictive value, and accuracy. Additionally, we determined the appropriate hemoglobin cut-off values for HemoPro and Rad-67 using receiver operating characteristic (ROC) analyses and the Youden index. Finally, we compared satisfaction levels between HemoPro and Rad-67 with the McNemar test.

## Results

**Fig 1:**
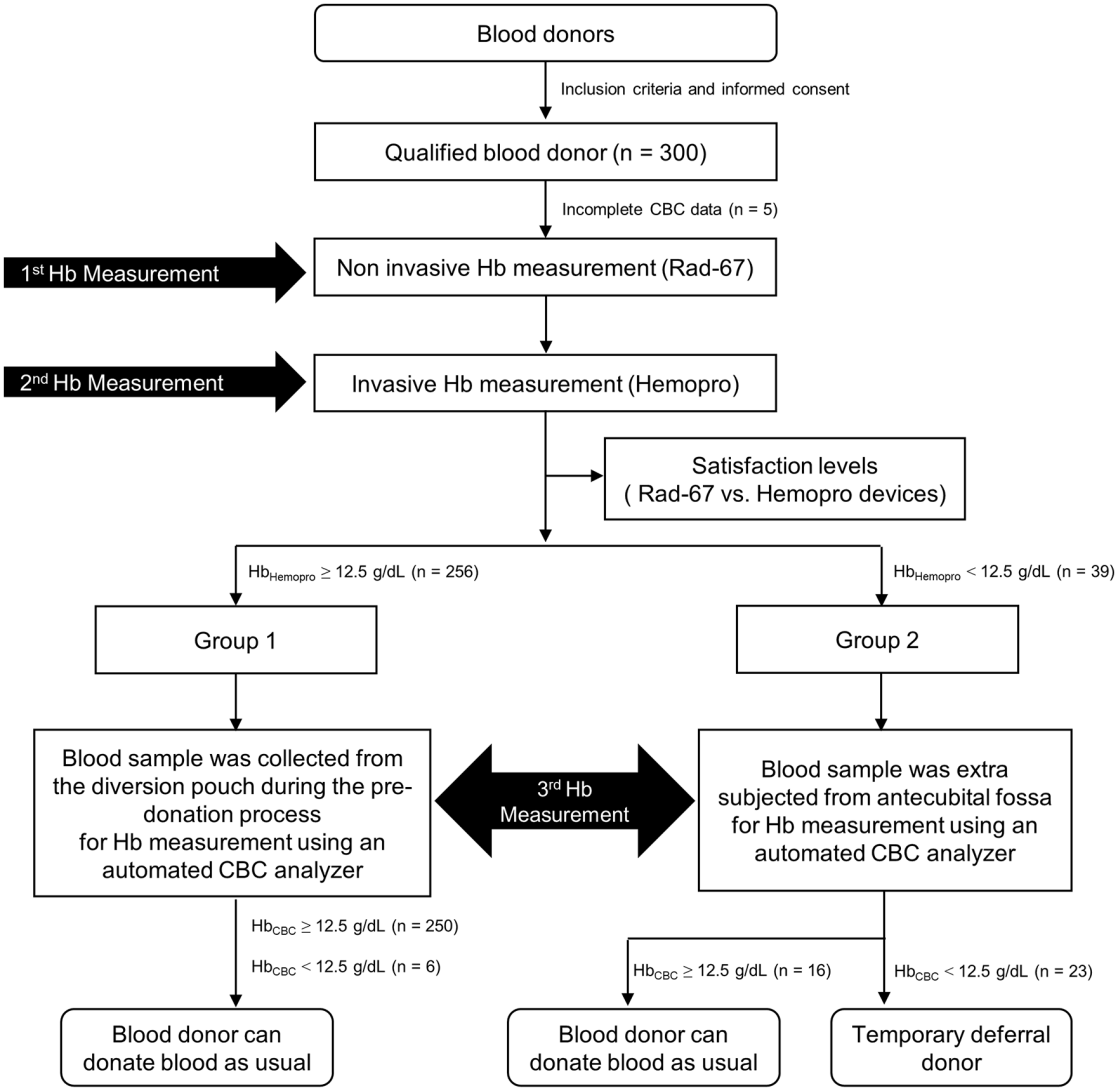
Number of blood donors and the result of hemoglobin measurement from three different methods.

Of 300 enrolled participants, 295 had complete data; 5 participants had incomplete CBC data. According to our institute#39;s criteria, the cut-off hemoglobin concentration for deferring blood donation is 12.5 g/dL. Using the point-of-care invasive device, the total number of participants who met the criteria (healthy donor group; Hb_Hemopro_ ≥ 12.5 g/dL) was 256 (164 males and 92 females). There were 39 participants (5 males and 34 females) who did not meet the criteria (temporary deferral group; Hb_Hemopro_ < 12.5 g/dL). Flow details of the study were listed in Fig 1.

As illustrated in Table 1, 169 (57.28%) were male. Average ages were 41.10 years for females and 41.92 years for males. Hemoglobin levels obtained from both the automated analyzer (Hb_CBC_) and from a non-invasive device (Hb_Rad-67_) were higher in male donors. Average Hb_Rad-67_ Hb_Hemopro_ and Hb_CBC_ were 14.38, 13.65, and 13.9 g/dL, respectively.

**Table 1:** Demographic data.

	Female	Male	Total	P-value
**Number (%)**	126 (42.71)	169 (57.28)	295 (100.00)	
**Age, years (mean ± SD)**	41.1 ± 10.4	41.9 ± 10.8	41.6 ± 10.6	0.512
**Hb level, g/dL (mean ± SD)**				
**Rad-67**	13.69 ± 0.82	14.89 ± 1.03	14.38 ± 1.12	<0.001
**HemoPro**	12.80 ± 0.42	14.29 ± 1.20	13.65 ± 0.70	<0.001
**CBC analyzer**	13.09 ± 0.73	14.51 ± 1.03	13.90 ± 1.16	<0.001

### Agreement assessment

The assessment of agreement regarding the reference Hb_CBC_ values revealed varying degrees of concordance among the tested methods. The ICC for Hb_Rad-67_ was 0.600, indicating moderate agreement, whereas Hb_Hemopro_ demonstrated a stronger correlation with an ICC of 0.897, reflecting good agreement.

**Fig 2:**
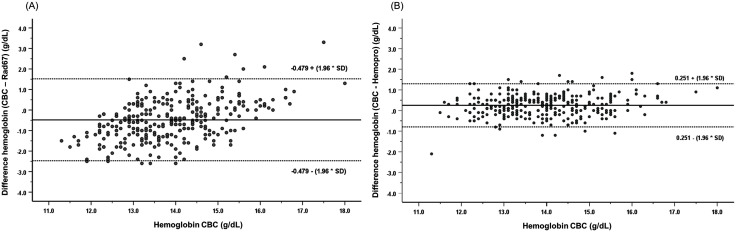
Bland-Altman plots illustrating the agreement between hemoglobin monitoring devices and the reference CBC method. (A) Difference between hemoglobin measurement by CBC and Rad-67 plotted against reference hemoglobin CBC values. (B) Difference between hemoglobin measurement by CBC and HemoPro plotted against reference hemoglobin CBC values. The solid horizontal lines represent the mean difference (bias), and the dotted lines indicate the 95% limits of agreement (mean ± 1.96 SD).

Bland-Altman analysis assessed the agreement between hemoglobin measurements and the reference CBC method (Fig 2). The Rad-67 device demonstrated a mean bias of −0.479 g/dL with 95% limits of agreement (LoA) ranging from −2.46 to 1.50 g/dL, indicating a systematic overestimation compared to the reference method (Fig 2A). Conversely, the HemoPro device exhibited a mean bias of 0.251 g/dL (95% LoA: −0.79 to 1.30 g/dL), reflecting a slight underestimation relative to CBC (Fig 2B). The HemoPro displayed narrower limits of agreement compared to the Rad-67, although the majority of data points for both devices fell within the 95% LoA.

### Sensitivity, specificity, and accuracy

According to the hemoglobin level obtained with the standard device (Hb_CBC_), the point-of-care HemoPro device correctly identified 250 participants, and Rad-67 correctly identified 261 of 266 participants with Hb_CBC_ ≥ 12.5 g/dL. However, HemoPro misclassified 6, and Rad-67 misclassified all 29 individuals with Hb_CBC_ < 12.5 g/dL. Thus, to identify donors with hemoglobin <12.5 g/dL, HemoPro demonstrated a sensitivity of 79.31%, specificity of 93.98%, a positive predictive value (PPV) of 58.97%, a negative predictive value (NPV) of 97.66%, and an overall accuracy of 92.54%. The Rad-67 showed a sensitivity of 0.00%, specificity of 98.12%, a PPV of 0.00%, and a NPV of 69.60%, with an overall accuracy of 68.68% compared with the CBC device (Table 2). When donors were classified by sex (Table 3), most donors with hemoglobin levels below 12.5 g/dL were female (25 of 29). The HemoPro could identify 23 of 29 donors with Hb < 12.5, but the non-invasive device failed to identify all donors with this condition.

**Table 2:** The number of participants whose Hb_Rad-67_ levels and Hb_Hemopro_ were ≥ 12.5 g/dL and <12.5 g/dL compared to the same Hb_CBC_ ranges.

		Hb_CBC_			
		< 12.5 g/dL	≥ 12.5 g/dL	Total	
Hb_Rad-67_	**< 12.5 g/dL**	0	5	290	Sensitivity 0.00% Specificity 98.12% Accuracy 68.68% PPV 0.00% NPV 69.60%
	**≥ 12.5 g/dL**	29	261	5	
	**Total**	29	265	295	
Hb_Hemopro_	**< 12.5 g/dL**	23	16	39	Sensitivity 79.31% Specificity 93.98% Accuracy 92.54% PPV 58.97% NPV 97.66%
	**≥ 12.5 g/dL**	6	250	256	
	**Total**	29	266	295	

PPV, positive predictive value; NPV, negative predictive value

**Table 3:** Evaluating the blood donor screening capability of the Rad-67 device by gender.

			CBC			
			Hb < 12.5 g/dL	Hb ≥ 12.5 g/dL	Total	
Rad-67	**Female**	**Hb < 12.5 g/dL**	0	2	2	Sensitivity 0.00% Specificity 98.02% Accuracy 68.61% PPV 0.00% NPV 69.58%
		**Hb ≥ 12.5 g/dL**	25	99	124	
		**Total**	25	101	126	
	**Male**	**Hb < 12.5 g/dL**	0	3	166	Sensitivity 0.00% Specificity 98.18% Accuracy 68.73% PPV 0.00% NPV 69.61%
		**Hb ≥ 12.5 g/dL**	4	162	3	
		**Total**	4	165	169	

PPV, positive predictive value; NPV, negative predictive value.

### Hb cut-off analysis

**Fig 3:**
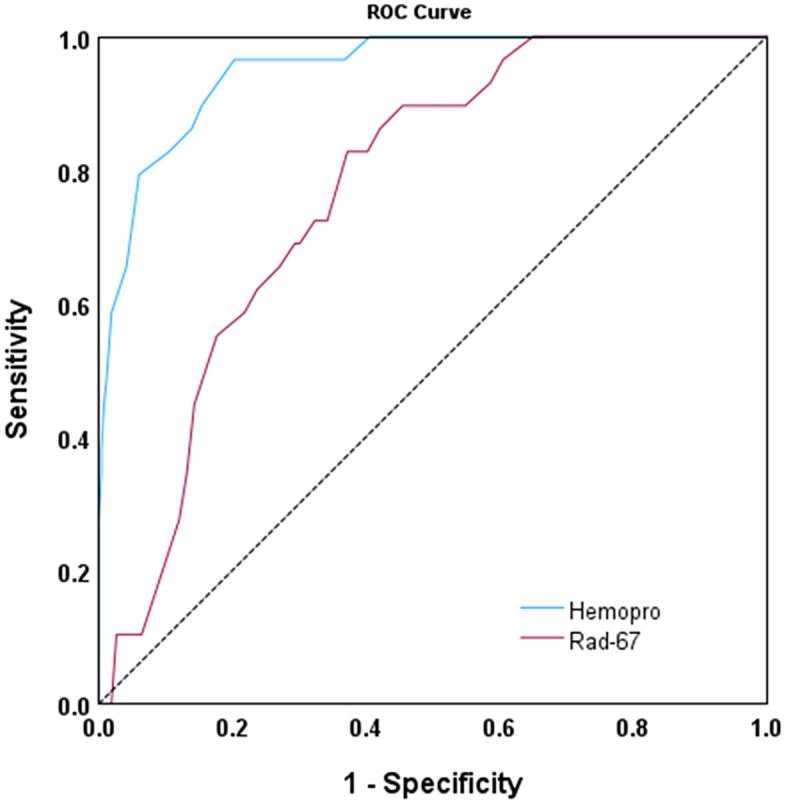
Receiver operating characteristic curves for the Hb cutoff point.

The ROC curve analysis (Fig 3) demonstrated that HemoPro exhibited high diagnostic performance, with an Area Under the Curve (AUC) of 0.951 (95% CI: 0.918–0.984, p < 0.001). In comparison, Rad-67 showed an AUC of 0.770 (95% CI: 0.699–0.842, p < 0.001). However, HemoPro displayed a trend towards higher overall sensitivity and specificity compared to Rad-67.

We evaluated the values of Hb_Hemopro_ and Hb_Rad-67_ against the reference Hb_CBC_ using the ROC curve and determined the optimal cut-off points for Hb_Hemopro_ at 12.85 g/dL and for Hb_Rad-67_ at 14.15.

The Youden index for HemoPro and Rad-67 at the cut-off value of 12.5 g/dL was 2.551 and 1.180, respectively. The highest Youden index of 2.729 for HemoPro was observed at a cut-off value of 12.8, while for Rad-67, it was 2.339 at a cut-off of 14.4.

Receiver operating characteristic (ROC) curves were constructed to determine the optimal Hb cut-off point.

### Satisfaction

The pairwise comparison of participant satisfaction between the Rad-67 and HemoPro devices across the domains of duration, pain, and safety using the McNemar test is shown in Table 4. Statistically significant differences were found in all three categories (p < 0.001). Analysis of the discordant pairs shows that, for duration, pain, and safety, a notably higher number of participants reported high satisfaction (Levels 4–5) with the Rad-67 while rating the HemoPro lower (Levels 1–3), compared to the opposite scenario. This indicates a clear preference for the Rad-67 device across all measured domains.

**Table 4:** Number of participants in three categories of satisfaction using the McNemar test.

Satisfaction levels		HemoPro		P-value
		Levels 1–3	Levels 4–5	
**Rad-67**	**Duration**			<0.001
	**Levels 1–3**	2 (0.68%)	8 (2.71%)	
	**Levels 4–5**	68 (23.05%)	217 (73.56%)	
	**Pain**			<0.001
	**Levels 1–3**	0 (0.00%)	3 (1.02%)	
	**Levels 4–5**	155 (52.54%)	137 (46.44%)	
	**Safety**			<0.001
	**Levels 1–3**	1 (0.34%)	3 (1.02%)	
	**Levels 4–5**	101 (34.24%)	190 (64.41%)	

## Discussion

The primary objective of this study was to evaluate the accuracy of the Rad-67 non-invasive hemoglobin measurement device compared to the standard automated CBC analyzer. However, the point-of-care test HemoPro was also included in the study and analysis because it is the current method for pre-donation hemoglobin screening. This was aimed at examining the possibility of using this test in the routine blood donor screening process. Main findings included moderate agreement between the tests, with a tendency for Rad-67 values to be higher. In addition, this non-invasive test failed to detect all donors with hemoglobin of 12.5 g/dL or lower, which is the routine deferring value for blood donation. The point-of-care hemoglobin measurement gave a better correlation and less bias with CBC result. Finally, compared with the portable hemoglobin analyzer, the Rad-67 device offers a less invasive and more comfortable experience for donors.

These findings mostly paralleled previous studies. A study of blood donors from Korea [12] compared a non-invasive device (NBM-200) different from ours with a point-of-care capillary hemoglobinometer (HemoCue) to the standard automated blood analyzer. Correlation between NBM-200 and the standard was substantial (ICC = 0.69), while that between HemoCue and the standard was 0.89. Hemoglobin measurement using NBM-200 showed a left-skewed distribution, indicating that the test tended to give higher hemoglobin results than the standard. A study in perioperative patients [15] compared the Rad-67 to the standard test. When using hemoglobin cut-off of 13 g/dL, poor performance in identifying anemia was noted. This was similar to our results. Additionally, when analyzing the female population, the screening accuracy was lower than that observed in males. A study from an antenatal setting in Kenya [9] also demonstrated a weak positive correlation between results from the Rad-67 and the standard tests with a proportional bias of 0.44 (95% CI: 0.41–0.47) and a constant of 7.59 (95% CI: 7.30–7.87, p < 0.001). The median bias was 1.70 g/dl, with limits of agreement of −0.80 to 4.20. Hemoglobin level obtained from the non-invasive device tended to be higher than Lab Hb in the low hemoglobin range but lower than Lab Hb in the high hemoglobin range. These studies and ours, in part, might be explained by the finding that higher hemoglobin levels were found in capillary blood than in venous blood [5]. However, when comparing the RAD-67 with HemoCue 301, which was a hand-held portable hemoglobin analyzer using capillary blood in non-pregnant women of reproductive age in Ethiopia, the results were the same [10]. Also, the Rad-67 showed lower sensitivity in detecting anemia (49%) than the HemoCue test. Finally, a study in blood donors from Korea, which compared a different non-invasive device to the standard automated blood analyzer showed moderate agreement [12]. All the above findings, including ours, suggest that the non-invasive spectrophotometric assessment of hemoglobin showed moderate agreement with the standard test. Across the board, sensitivity to detect anemia was low.

Based on the information above, we can answer whether the Rad-67 device can be used to screen blood donors. Obviously, the device failed to identify individuals with Hb levels below 12.5 g/dL. These findings indicated that the Rad-67 had limited effectiveness in detecting low Hb levels. Even in the study that used an Hb cutoff of 13 g/dL [15], the accuracy was very low. Hence, with these cut-off values, the Rad-67 could not be used to defer donors with suspected anemia. Given that the donors preferred a non-invasive method, we may adjust the cut-off values to improve diagnostic accuracy. When focusing on negative predictive value, which was the acceptance point for donation, Hb of 13.5–14.5 gave substantial numbers. Thus, an alternative approach to avoid painful screening in most donors is to increase the cut-off value to 14.4, as indicated by the ROC and the Youden index. Only individuals with non-invasive Hb below that value will be screened with a more accurate method. This approach may avoid invasive hemoglobin test in 50–60% of blood donors.

The strengths of this study included random selection of blood donors and the comparison of the new device with a standard reference, which helped minimize selection bias. Hemoglobin was measured in the same healthy individuals to reduce the impact of demographic differences that could affect Hb values.

Our study had several limitations. First, we did not evaluate the reproducibility of each non-invasive hemoglobin measurement by the same or different operators at different times. Additionally, we did not document the characteristics of the donor’s finger, which could influence the accuracy of the non-invasive device#39;s measurements.

## Conclusion

In summary, although the Rad-67 device showed limited agreement with the automated CBC analyzer and was not sensitive for detecting low hemoglobin levels, blood donors preferred it over the more accurate invasive point-of-care hemoglobin test. Raising the Hb cut-off threshold improves diagnostic accuracy. Half of the blood donors might avoid the invasive hemoglobin measurement, making blood donation a more pleasant experience. Further study is needed to prove this postulation in a larger population.

## Supporting information

S1 File Supporting data file for “Evaluation of non-invasive hemoglobin measurement in blood donors at a tertiary care hospital, Bangkok, Thailand”. (XLSX)
